# Prefrontal Single-Neuron Responses after Changes in Task Contingencies during Trace Eyeblink Conditioning in Rabbits

**DOI:** 10.1523/ENEURO.0057-16.2016

**Published:** 2016-07-18

**Authors:** Jennifer J. Siegel

**Affiliations:** Center for Learning and Memory and the Department of Neuroscience, The University of Texas at Austin, Austin, Texas 78712

**Keywords:** classical conditioning, executive function, persistent activity, prefrontal cortex, trace conditioning, working memory

## Abstract

A number of studies indicate that the medial prefrontal cortex (mPFC) plays a role in mediating the expression of behavioral responses during tasks that require flexible changes in behavior. During trace eyeblink conditioning, evidence suggests that the mPFC provides the cerebellum with a persistent input to bridge the temporal gap between conditioned and unconditioned stimuli. Therefore, the mPFC is in a position to directly mediate the expression of trace conditioned responses. However, it is unknown whether persistent neural responses are associated with the flexible expression of behavior when task contingencies are changed during trace eyeblink conditioning. To investigate this, single-unit activity was recorded in the mPFC of rabbits during extinction and reacquisition of trace eyeblink conditioning, and during training to a different conditional stimulus. Persistent responses remained unchanged after full extinction, and also did not change during reacquisition training. During training to a different tone, however, the generalization of persistent responses to the new stimulus was associated with an animal’s performance—when persistent responses generalized to the new tone, performance was high (>50% response rate). When persistent responses decreased to baseline rates, performance was poor (<50% response rate). The data suggest that persistent mPFC responses do not appear to mediate flexible changes in the expression of the original learning, but do appear to play a role in the generalization of that learning when the task is modified.

## Significance Statement

The medial prefrontal cortex (mPFC) plays a role in executive function, controlling the expression or inhibition of behaviors. But it is not clear under what conditions such executive control is observed, or how neural activity in the mPFC might mediate the expression of behavior. Trace eyeblink conditioning is known to rely on mPFC neurons responding persistently to a training cue, and offers an opportunity to test whether changes in the activity of these neurons might mediate changes in behavior when task contingencies are altered. PFC cells continued to respond persistently after extinction training, suggesting that the mPFC may not mediate the inhibition of previous learning. However, the generalization of persistent responses to a new task was associated with successful performance.

## Introduction

The ability to show flexible changes in learned behavior based on new experience is critical for survival. In mammals, this kind of “executive function” is often attributed to the medial prefrontal cortex (mPFC), and various disease states involving this structure can result in an inability to show flexible learning and the perseveration of previously learned behaviors (Kolb, 1990; Miller, 2000). Tasks that directly rely on the mPFC are particularly suitable for investigating how changes in neural activity may be associated with adaptive changes in behavior.

**Figure 1.**
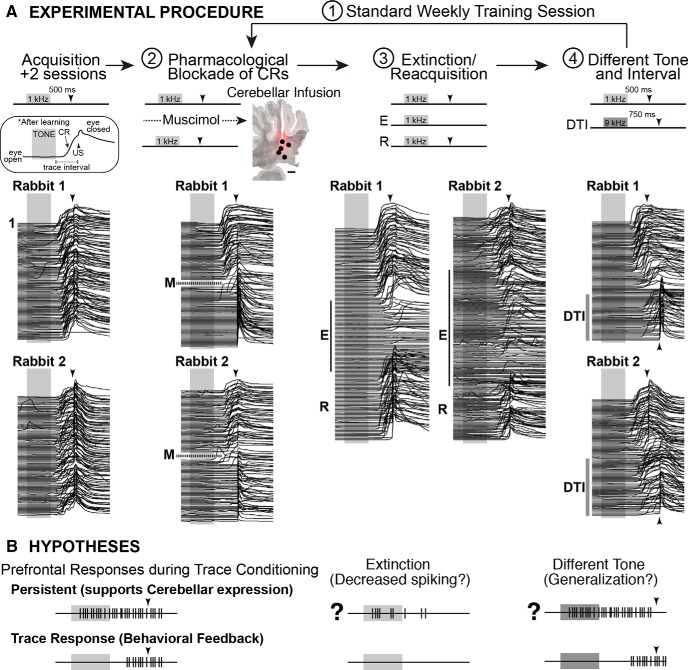
Outline of experimental procedures, example behavior, and schematic of hypotheses regarding how mPFC cells may alter responses and mediate behaviors as a result of flexible learning. ***A***, Rabbits (*n* = 8) received standard training for trace eyeblink conditioning until asymptotic. Inset shows the standard training protocol and example eyelid response after learning (upward deflection indicates closure). Waterfall plots show traces of eyelid position for each trial of a session for two representative rabbits (trial 1 is at top, *x*-axis is time: gray bar, 500 ms tone; arrowhead, US onset). Eyelid closures prior to US onset are CRs. After acquisition, rabbits experienced four types of training sessions each week: Day 1, standard training; Day 2, pharmacological blockade of CRs (M, muscimol infusion into the cerebellum; note absence of CRs after infusion for both example sessions; markers show histologically verified infusion sites around Bregma −19.5; scale bar, 1 mm); Day 3, tone-only extinction training (E) followed by reacquisition (R; note the decrease in CRs during E training and the fast reinstatement of behavior during R training); and Day 4, different tone (9.6 kHz) and interval training (750 ms DTI; note the temporal shift in CR onsets during DTI). After Day 4, recording tetrodes were advanced to isolate a different population of mPFC cells, with daily manipulations repeated the following week. ***B***, Schematic of persistent and “trace” (CR feedback responses) single-unit spike responses observed in the mPFC during trace conditioning (left) and the hypothesized response changes if persistent cells mediate flexible learning.

The mPFC is critical for the acquisition and ongoing expression of trace eyeblink conditioning (TEC; Kronforst-Collins and Disterhoft, 1998; Kalmbach et al., 2009; Chen et al., 2014; Siegel et al., 2015). In TEC, an initially neutral conditional stimulus (CS; e.g., a tone) predicts the occurrence of an unconditional stimulus (US; e.g., an air puff to the eye causing reflexive eyelid closure). The stimuli are separated by a stimulus-free delay interval (the “trace interval”; Fig. 1*A*, top left). After training, the animals learn to close the eyelid whenever the CS is presented [conditional response (CR); Fig. 1*A*, top left]. The putative role of the mPFC in TEC is to provide a persistent input to the cerebellum (via the pons) that bridges the trace interval to overlap with the US and enable cerebellar motor learning (Fig. 1*B*, left; Takehara-Nishiuchi and Mcnaughton, 2008; Kalmbach et al., 2009; Weiss and Disterhoft, 2011; Siegel et al., 2012; Siegel and Mauk, 2013; Chen et al., 2014; Hattori et al., 2014; Siegel, 2014). Because the mPFC is necessary not just for acquisition, but also for the ongoing expression of trace CRs (Takehara et al., 2003; Kalmbach et al., 2009; Siegel and Mauk, 2013; Chen et al., 2014; Siegel et al., 2015), this brain region is in an ideal position to mediate behavioral responses based on new learning. Whether or not a CR is expressed could be controlled directly by altering the output of mPFC neurons providing input to the cerebellum.

The goal of the current study was to determine whether changes in the persistent responses of mPFC cells are associated with the flexible expression of learned behavior. To this end, we recorded mPFC cells during the extinction and reacquisition of trace CRs, and during training to a different CS (Fig. 1). The responses of persistent cells were not altered during extinction or reacquisition, suggesting that learning not to respond may occur downstream from the mPFC or in a different mPFC region. When training to a different tone, however, persistent mPFC neurons showed generalized CS responses that were associated with the performance of an animal during the new conditions. The data reveal that the mPFC can generalize previously learned associations when task contingencies are changed and support adaptive behavior based on new experience.

## Materials and Methods

### Subjects and surgical procedures

All surgical and experimental procedures were approved by the University of Texas at Austin Institutional Animal Care and Use Committee and were in accordance with the National Institutes of Health guidelines. Eight New Zealand albino rabbits (males; weight, 2.5–4 kg; Myrtle's Rabbitry Inc) were implanted with custom-built microdrives housing 18 independently moveable tetrodes targeting the caudal anterior cingulate/medial agranular regions of mPFC, shown previously to play a role in the expression of trace eyeblink conditioning (Kronforst-Collins and Disterhoft, 1998; Powell et al., 2001; Kalmbach et al., 2009; Chen et al., 2014). For surgical preparation, each rabbit was given a subcutaneous injection of ketamine (45 mg/kg) and acepromazine (1.5 mg/kg), and mounted in a specialized stereotaxic apparatus (with λ 1.5 mm below bregma). Isoflurane gas (1–3% in medical grade oxygen) was used to maintain surgical depth anesthesia levels. Microdrive tetrode bundles (1.5–2.5 mm in diameter) were positioned on the surface of the brain over the right posterior mPFC (centered at 3.0 mm anterior to bregma and 1.0–1.5 mm lateral from the midline), and secured to the skull with screws and dental cement. Each rabbit was also prepared with a head bolt fixed in dental cement over the anterior skull to hold the eyeblink detector during conditioning. For periorbital stimulation, two stainless steel stimulating electrodes were implanted subdurally just anterior and posterior to the upper eyelid contralateral to the microdrive. Each rabbit was allowed at least 1 week of recovery before training began.

### Standard behavioral training and analysis

Training used a standard trace eyeblink conditioning protocol as previously described (Siegel et al., 2012; Siegel and Mauk, 2013; Siegel, 2014). Rabbits were gently restrained and placed in a shielded, sound-attenuating chamber (Gormezano et al., 1983; Kalmbach et al., 2009; Siegel et al., 2012). Daily training sessions were controlled by custom software and consisted of 12 blocks of 9 trials for a total of 108 trials per session. The first trial of every block was a CS-only probe. Trials were presented at random intertrial intervals drawn from a flat distribution between 25 and 35 s (intervals between the last and first trials between blocks were the same as within-block trial intervals). The CS consisted of a 500 ms 1.3 kHz pure tone with rise and fall times of 5 ms (to avoid audible onset and offset transients). The US consisted of a 50 ms train of current pulses (1 ms pulse width at 100 Hz) delivered across the periorbital electrodes. The US intensity was carefully adjusted for each rabbit to just above threshold to elicit a full eyeblink closure (between 1 and 3 mA). On paired training trials, the CS was followed by a 500 ms stimulus-free period (the trace interval) and terminated with US presentation (Fig. 1*A*, top left). For each trial, the position of the external eyelid was measured using an infrared emitter and collector assembly mounted on the head bolt. Closure of the eyelid resulted in an increased amount of reflected infrared light, which was detected with an infrared detector and converted to a voltage deflection. Eyelid behavior was sampled at 1 kHz for 2.5 s, beginning 200 ms prior to CS onset, and stored for off-line analysis. Before each training session, the eyeblink detector was calibrated by measuring the voltage deflection produced by a full eyelid closure and defining that voltage change as 6.0 mm (the amplitude of a full eyelid closure in a rabbit). A CR was defined as an eyelid response that exceeded 0.3 mm between CS and US onsets (Mauk and Ruiz, 1992; Garcia et al., 1999; Kalmbach et al., 2009; Siegel et al., 2012).

Rabbits were given one training or manipulation session per day. The likelihood of CRs was calculated for each training condition within a session by dividing the number of trials in which a CR was observed by the total number of trials for that condition. Rabbits were considered to have met the learning criterion when they displayed the first instance of eight CRs in nine consecutive trials. For initial acquisition, this criterion always occurred in the initial session in which total CR likelihood for the session exceeded 50%. Once the criterion was met, rabbits were trained for a minimum of two additional sessions to establish asymptotic performance before proceeding to behavioral manipulations.

### Weekly training procedure

After acquisition (see above), rabbits experienced behavioral manipulations once per week, as outlined in Figure 1*A*. Day 1 was a standard training (Std) session to serve as a control. Day 2 used pharmacological blockade of the expression of CRs by infusion of muscimol into the anterior deep cerebellar nuclei (see below). Day 3 began with standard training for three to six blocks, followed by CS-only extinction (six or more blocks, depending on the behavior of the animal) and concluded with reacquisition training (reinstatement of standard training). Day 4 began with standard training for six blocks, followed by training to a different tone CS (9.6 kHz) and a longer trace interval (from 500 to 750 ms, see below). Behavior during standard training each day did not appear to be affected by the manipulation experienced the previous day (representative behavioral sessions for a given week from two rabbits are shown in Fig. 1*A* for each type of manipulation). At the conclusion of the different tone/interval training on day 4, recording electrodes were lowered 160 µm to isolate a new population of mPFC cells, and the manipulations were repeated after a 3 d wait to allow recordings to stabilize. Weekly procedures were repeated for a given rabbit until more than half of the tetrode tips reached the ventral extent of the mPFC and entered the corpus callosum (3–18 weeks, depending on the placement of the tetrode bundle in the mediolateral axis and the depth of tetrodes at the start of the experiments).

### Pharmacological blockade of CR expression in the cerebellum

To temporarily block the expression of CRs in the absence of learning, a 1 mm solution of muscimol (Tocris Bioscience) dissolved in artificial cerebrospinal fluid (in mm: 119.0 NaCl, 2.5 KCl, 1.2 NaH_2_PO_4_, 26.0 NaHCO_3_, 2.0 CaCl_2_, 2.0 MgCl_2_, 10.0 dextrose, 10.0 HEPES; pH adjusted to between 7.35 and 7.4, and passed through a 2 μm filter to sterilize) was infused into the anterior deep cerebellar nuclei (Fig. 1*A*, cerebellar infusions). After three to six blocks of standard training, the session was paused and muscimol infused (0.2 μl/min, 2 μl total volume). Training resumed 5 min after completion. An infusion was considered effective when the likelihood of CRs decreased below 50% for a given block, followed by <15% CRs observed for all remaining blocks. Most rabbits showed an immediate and complete behavioral effect upon resuming the session that lasted for the remainder of the session (Fig. 1*A*, pharmacological blockade of CRs).

### Extinction and reacquisition sessions

Extinction and reacquisition were conducted within the same recording sessions to ensure that the responses of a given neuron could be tracked across all behavioral conditions. Previous work showed that extinction training using CS-only trials is the most efficient method, and so that procedure was implemented here (Kehoe, 2006). Each session began with 3–6 blocks of standard training, followed by CS-only trials until full extinction was observed (6–12 blocks; the criterion was 8 of 9 trials with no CR), and then concluded with reacquisition training (3–6 blocks; Fig. 1*A*, extinction/reacquisition). In 5 of 45 extinction sessions, rabbits did not receive reacquisition training. Analyses of single-unit responses were made between standard training (the last 27 trials before extinction training began) and after full extinction (the first 27 trials after the criterion was met). For reacquisition, spike analysis was performed on the first 27 trials after a criterion of 8 of 9 trials with a CR was met. Some rabbits did not meet reacquisition criteria before the maximum number of trials allowed for a single session (maximum number of trials/day, 216), but often showed evidence of reinstatement even when criterion was not met (CR rates between 30% and 75%). Spike data from these sessions (24 of 40 sessions) were analyzed separately (see Results) and used the last 27 trials of reacquisition for analysis.

### Different tone and trace interval training and behavioral analysis

To examine whether mPFC cells show generalized responses to a different CS that could support flexible behavior, it is critical to show that a given rabbit did not simply continue to make the originally learned behavioral response to the different CS. Therefore, a longer trace interval was used in association with the different tone (from 1.3 to 9.6 kHz, and from 500 to 750 ms). Only sessions in which rabbits showed significant shifts in the timing of behavioral responses specific to each tone were included in analyses (based on latencies to CR onset, data from 33 of 40 sessions met this criterion). Different tone/interval training sessions were composed of three to five blocks of standard training followed by one to two blocks of CS-only extinction (to reduce the likelihood of CR expression), followed by four to six blocks of different tone/interval training (Fig. 1*A*, different tone and interval). Preliminary work indicated that the inclusion of one to two blocks of extinction before different tone training increased the likelihood that rabbits would differentiate behavioral responses between the two tones. Epochs for spike activity analyses were based on the last 36 trials of standard training and the last 36 trials of different tone training.

### Single-unit recordings

Neural activity was acquired with a Digital Lynx system (Neuralynx). Tetrodes were constructed from polyimide-coated nichrome wire (12 μm diameter; Kanthal Palm Coast) and gold plated to an impedance of 0.5–1.25 MΩ at 1 kHz (postimplantation impedances, 0.7–2.0 MΩ). Tetrode signals were passed through a multichannel unity-gain headstage, amplified, bandpass filtered between 600 and 6000 Hz, and then digitized at 32 kHz for off-line spike sorting and analyses. Neural data were synchronized with the presentation of training stimuli by triggering the Digital Lynx I/O port with the same transistor–transistor logic pulses used to trigger CS and US stimuli.

**Figure 2.**
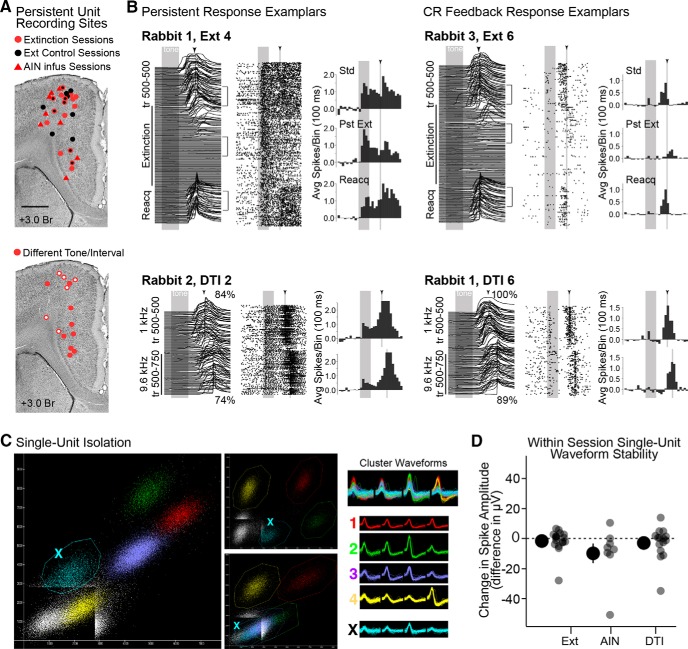
Estimated recording sites of persistent cells, example single-unit responses, single unit cluster isolation and stability measurements for manipulation sessions. ***A***, Top, Estimated recording locations of persistent mPFC neurons recorded during extinction (red circles) and control extinction sessions (black circles; markers outlined in red indicate persistent cells recorded at that location for both control and corresponding extinction sessions). Recordings during cerebellar infusions (AIN) are shown as red triangles. Bottom, Recording sites for DTI sessions. Open markers indicate recordings during sessions with poor performance, closed markers indicate good performance. ***B***, Waterfall plots show eyelid behavior. Raster plots show spike data across trials (each row is a trial; each dot is a spike; gray bars and arrowheads indicate the presentation of tone and US, respectively). Brackets indicate trials used for spike analysis based on behavioral criteria (see Materials and Methods), and histograms (baseline subtracted) show averaged spike data from those epochs (Std; Pst, postextinction; Reacq, reacquisition training). Top examples are from extinction sessions; bottom examples are from different tone/interval sessions. ***C***, Scatterplots showing different peak-to-peak comparisons from two of the four channels of a tetrode. Colors indicate putative isolated single-unit clusters, with corresponding waveforms given to the right. Note that the teal cluster never completely surpasses the trigger threshold, while other clusters do and show clean separation from each other on at least one projection. The teal cluster would be eliminated from analyses due to poor isolation. ***D***, Isolated clusters showing persistent responses during Ext, cerebellar infusion (AIN), and DTI training were evaluated for stability within recording sessions. Large markers show the mean and SEM of changes in amplitude between standard and experimental trials (individual data points are also shown). The peak spike amplitudes of some persistent cells shifted during recording sessions, but no systematic changes were observed for any group (*t* = 0.79–1.5, *p* > 0.05, n.s.).

The activity of single units was isolated off-line using interactive cluster-cutting software (WinClust; adapted from MA Wilson), as previously described (Siegel et al., 2008, 2012; Siegel 2014). Isolated clusters of suprathreshold neural events with common waveform parameters were identified as single units, and the timestamps of those events (spikes) extracted and analyzed relative to the presentation of training stimuli and behavioral responses. Only single units with clustered points that showed little or no overlap (<10%) with another cluster or with background activity were included for analysis (Fig. 2*C*; Siegel et al., 2008, 2012). Cluster stability during sessions was assessed for persistent cells by calculating the average peak amplitude observed during the last 27 s (three blocks) of standard training and the average observed for experimental trials included for analysis (based on channels with the largest spike peak; range, 47–225 µV; mean, 116.9 ± 5.1 µV). Significant decreases or increases in amplitude were sometimes observed, particularly for larger spikes, but systematic changes that could have influenced the results were not observed for any of the comparisons^a^ [Fig. 2*D*; i.e., the average change for each experimental group was not different than 0 for Ext, anterior interpositus nucleus (AIN) infusion, and different tone/interval (DTI), respectively: *t* = 0.79, 1.5, 1.05; df = 16, 7, 14; *p* = 0.44, 0.18, 0.31; Table 1].

**Table 1:** Statistics

	Data structure	Type of test	Groups compared	Confidence intervals
**a**	Unknown	Paired *t* test, two-tailed	Pre- vs post-spike height: Ext, AIN infusion, DTI	95% (each comparison)
**b**	Unknown	Spearman correlation (with Bonferroni correction)	Spiking of each cell with corresponding eyelid responses	99% (corrected for 3 bins = 99.7% CI each bin)
**c**	Unknown	Bootstrap (with Bonferroni correction)	Pre- vs post-Ext: eyelid responses	99% (corrected for 10 bins = 99.9% CI each bin)
**d**	Unknown	Bootstrap (with Bonferroni correction)	Pre- vs post-Ext: trace cells	99% (corrected for 10 bins = 99.9% CI each bin)
**e**	Unknown	Bootstrap (with Bonferroni correction)	Pre- vs post-Ext: persistent cells	99% (corrected for 10 bins = 99.9% CI each bin)
**f**	Unknown	Bootstrap (with Bonferroni correction)	Pre- vs post-muscimol: persistent cells	99% (corrected for 10 bins = 99.9% CI each bin)
**g**	Unknown	Bootstrap (with Bonferroni correction)	Post-Ext vs muscimol: persistent cells	99% (corrected for 10 bins = 99.9% CI each bin)
**h**	Unknown	Bootstrap (with Bonferroni correction)	Pre- vs post-Ext: weak feedback persistent cells	99% (corrected for 10 bins = 99.9% CI each bin)
**i**	Unknown	Paired *t* test, two-tailed	Pre- vs post-Ext: number of coactive pairs	95%
**j**	Unknown	Paired *t* test, two-tailed	Ext and control: cell pair correlations (*r*)	95% (each comparison)
**k**	Unknown	Unpaired *t* test, two-tailed	Ext and control: change in *r* values	95%
**l**	Unknown	Bootstrap (with Bonferroni correction)	Ext vs reacqired: persistent cells	99% (corrected for 10 bins = 99.9% CI each bin)
**m**	Unknown	Bootstrap (with Bonferroni correction)	Ext vs reacquired: trace cells	99% (corrected for 10 bins = 99.9% CI each bin)
**n**	Normal	Paired *t* test, one-tailed	Tr500 vs Tr750: CR onset	95% (each session)
**o**	Normal	Paired *t* test, one-tailed	Tr500 vs Tr750: median CR onsets	95%
**p**	Unknown	Wilcoxon signed rank	Session 1 Tr500 vs session 1 Tr750: CR rate	95%
**q**	Unknown	Wilcoxon signed rank	Sessions 1 vs 2 Tr750: CR rate	95%
**r**	Unknown	Wilcoxon signed rank	Sessions 2 vs 3 Tr750: CR rate	95%
**s**	Unknown	Bootstrap (with Bonferroni correction)	Tr500 vs Tr750, >50% CRs: Persistent cells	99% (corrected for 10 bins = 99.9% CI each bin)
**t**	Unknown	Bootstrap (with Bonferroni correction)	Baseline spike rate vs trial spike rate, Tr750 >50% CRs: persistent cells	99% (corrected for 10 bins = 99.9% CI each bin)
**u**	Unknown	Paired *t* test, one-tailed	Tr500 vs Tr750: median CR onsets	95%
**v**	Unknown	Paired *t* test, one-tailed	Tr500 vs Tr750: median CR onsets	95%
**w**	Unknown	Bootstrap (with Bonferroni correction)	Tr500 vs Tr750, <50% CRs: persistent cells	99% (corrected for 10 bins = 99.9% CI each bin)
**x**	Unknown	Bootstrap (with Bonferroni correction)	baseline spike rate vs trial spike rate, Tr750 <50% CRs: persistent cells	99% (corrected for 10 bins = 99.9% CI each bin)
**y**	Unknown	Bootstrap (with Bonferroni correction)	Tr500 vs Tr750, >50% CRs: weak feedback, persistent cells	99% (corrected for 10 bins = 99.9% CI each bin)
**z**	Unknown	Bootstrap (with Bonferroni correction)	Tr750 >50% CRs vs Tr750 <50% CRs	99% (corrected for 10 bins = 99.9% CI each bin)
**a'**	Unknown	Bootstrap (with Bonferroni correction)	Tr500 >50% CRs vs Tr500 <50% CRs	99% (corrected for 10 bins = 99.9% CI each bin)
**b'**	Unknown	Bootstrap (with Bonferroni correction)	Tr500 vs Tr750, >50% and <50% CRs: phasic cells	99% (corrected for 10 bins = 99.9% CI each bin)
**c'**	Unknown	Bootstrap (with Bonferroni correction)	Tr500 vs Tr750, >50% and <50% CRs: CR feedback cells	99% (corrected for 10 bins = 99.9% CI each bin)

CI, Confidence interval; Tr500, Std for 500 ms; Tr750, Std for 750 ms.

### Histological procedures

At the conclusion of the experiments, rabbits received marker lesions through a subset of tetrodes (2 mA for 15–20 s). Rabbits were killed 48–72 h later by intravenous injection of Euthasol (0.3 ml/kg) and perfused intracardially with 0.9% saline, followed by 4% paraformaldehyde. The brains were extracted and postfixed in 4% paraformaldehyde or 10% formalin for 1–4 weeks before being cryoprotected in a 30% sucrose solution. The mPFC was sectioned on a freezing microtome (40–50 µm). Sections were mounted on Superfrost Plus slides (Fisher Scientific), dried overnight, then stained with cresyl violet to visualize tetrode tracks and surrounding anatomical structures. Approximate recording locations were determined by the amount of tetrode advancement between the last recording and the session during which the unit was recorded, and measuring that distance up from the location of the tetrode tip, which was identified histologically. Recording sites were plotted on a representative section of tissue based on location relative to surrounding landmarks (e.g., cortical layers, corpus callosum, brain surface, ventricle; Fig. 2*A*). The same histological procedures were used to determine the location of the cerebellar infusion cannula (Fig. 1*A*).

### Categorization of single-unit responses

For each isolated single unit, detected spikes were assigned to 100 ms time bins, beginning 1 s before CS onset and extending 1 s after US onset (Siegel et al., 2012; Siegel and Mauk 2013; Siegel, 2014). The mean number of spikes per bin observed for the 10 bins prior to CS onset was used as the baseline activity for that trial. The statistical reliability of changes in spike activity for each time bin (five bins during the CS and five bins during the trace interval) was determined with a paired *t* test between the number of spikes observed for that time bin and the average observed for 10 pre-CS time bins (with Bonferroni correction for the number of time bin comparisons, α = 0.01/10 trial time bins, *p* < 0.001). Single units were then categorized according to the pattern of significant changes in activity during trace conditioning trials, and whether the spike activity significantly increased or decreased relative to the pre-CS baseline (Siegel et al., 2012; Siegel and Mauk, 2013; Siegel, 2014). “Persistent” responses were defined as a significant change in spike activity that began before the end of the CS and persisted at least two bins (200 ms) into the trace interval, based on the minimum input necessary to engage cerebellar learning (Kalmbach et al., 2009; Siegel et al., 2012). The majority of persistent cells, however, showed spike responses that persisted across the entire trace interval and sometimes for seconds post-US (Figs. 1*B*, top left schematic, 2*B*, examples; Siegel et al., 2012). “Phasic” responses to the CS began during the CS but failed to meet persistent criteria. A third trace interval response type was defined by increased activity after CS offset and before US onset. This response was previously shown to typically reflect behavioral feedback regarding the CR (Siegel and Mauk, 2013; Fig. 1*B*, bottom left schematic), and is used in the current study as a readout of CR feedback to the mPFC.

### Identification of single units showing behavioral feedback

Previous work demonstrated that the spike activity of a subset of mPFC cells during trace eyeblink conditioning reflect behavioral feedback regarding the CR (Siegel and Mauk, 2013). The goal of the current study was to compare single-unit activity before and after flexible learning, based on changes in behavior. To control for apparent changes in spike response that could be due to changes in behavioral feedback and not upstream changes in the responses of mPFC cells to the CS, an analysis was developed to identify mPFC cells showing “strong” or “no/weak” CR-associated spike responses so that these cell types could be analyzed separately. CRs are predominantly observed during the last 300 ms of the trace interval, and show natural variability in latencies to onset and in amplitudes between trials within a session (representative behavior shown in Fig. 1*A*; see also Figs. 1A, 2B, 4D and 7A). Therefore, for each single unit a significant correlation between eyelid position and spike activity for any of the last three trial time bins (100 ms/bin) indicated that a neuron showed significant (“strong”) modulation of spike activity by behavioral feedback^b^ (Spearman Rank Correlation with Bonferroni correction for number of bins, alpha = 0.01, *p* < 0.003/bin). This analysis was cross-validated by comparing the responses of trace and persistent cells before and after pharmacological blockade of the expression of CRs during standard training. Cells identified as showing strong feedback also showed reliable changes in the last three trial time bins during CR blockade, suggesting that the cells were indeed modulated by behavioral feedback, while cells identified as showing no/weak behavior-associated spike activity did not show reliable changes during CR blockade.

**Figure 3.**
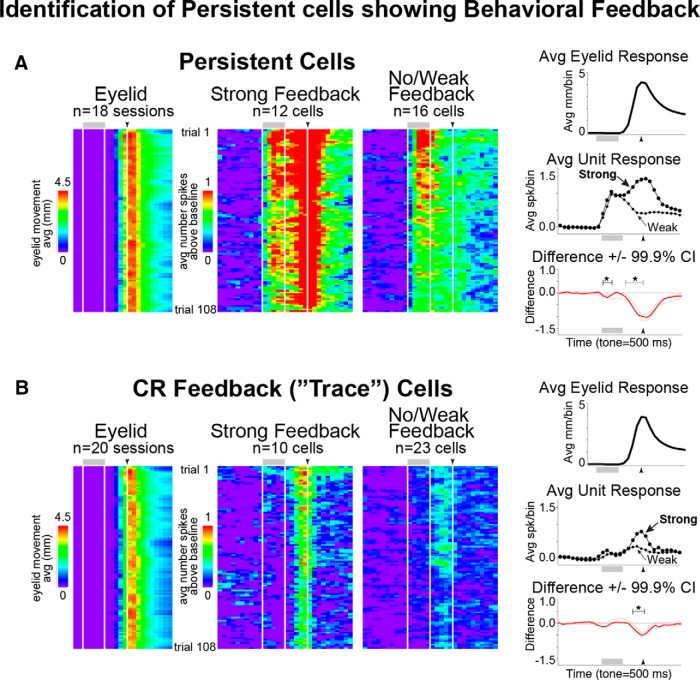
Comparison of mPFC cell types showing “Strong” or “No/Weak” behavioral feedback during standard training sessions. Cells receiving feedback were identified by correlating spike activity across trials for a given session with eyelid responses (see Materials and Methods). Pseudocolored matrices are binned, baseline subtracted, and averaged eyelid (left) or single-unit spike data (middle and right) from standard training sessions (each row represents a trial; at top, gray bars and arrowheads, with corresponding white lines, indicate presentation of tone and US, respectively). Graphs to right show session-averaged eyelid (top) and spike data for cells receiving strong or no/weak feedback (middle), and the bin-by-bin differences in spike rates (bottom, red line) and confidence intervals based on bootstrapping (red dashed lines; see Materials and Methods). ***A***, ***B***, Cells receiving strong or no/weak feedback differed most in time bins corresponding to the timing of behavioral responses for both persistent (***A***) and CR feedback (***B***) cell types, while responses during the tone were not substantially different. Avg, average.

### Bootstrap procedures to compare spike responses between training conditions

The general bootstrap procedures used here have been described previously (Siegel, 2014). In short, to test for changes in spike activity that may have occurred between behavioral conditions, for each single unit the spikes observed for each trial of a session were binned (100 ms). The resulting time bin × trial matrix of each cell was baseline subtracted and smoothed by a single pass with a 3 × 3 median filter. The filter was applied separately to pretrial bins, to bins from CS onset to US onset, and then to post-trial bins to guard against any contamination of activity during the CS and trace intervals from activity before or after these intervals. For each comparison (e.g., between standard training and full extinction, as epoch 1 vs epoch 2), the single-session matrices of all cells for a given categorical response type were averaged, such that each row of the averaged matrix represented the average of all cells for that trial number, and a difference score calculated for each time bin. A bootstrapping procedure was used to test whether time bin differences were reliably different than zero. For each iteration (×1000), the first epoch of the unsmoothed matrix of each cell was sampled with replacement, and the second epoch of the unsmoothed matrix of each cell was also sampled with replacement. The resampled matrices of each cell were smoothed in the same fashion as the original data, averaged, and then the difference scores for each time bin were calculated and stored. A confidence interval was calculated from the distribution of resampled difference scores for each time bin (α = 0.01/10 trial bins as a Bonferroni correction). Time bins for which zero fell outside the confidence interval were considered to show difference scores that were reliably different than zero (i.e., were significant at *p* < 0.001). The same procedure was used to test whether eyelid responses showed reliable changes due to training manipulations. Eyelid responses were binned (100 ms) into session matrices (time bin × trial), and restructured and analyzed in the same fashion as spike data to validate behavioral criteria.

### Population coactivity correlations

Changes in the coactivity of cell pairs were determined for simultaneously recorded cell ensembles. Raster plot matrices were made using 10 ms time bins for each cell in a given ensemble. A Pearson *r* value was first calculated for each cell pair for the initial 700 ms (70 time bins) across standard trials, and then repeated across the same postextinction trials used for previous analyses (the last 300 ms of each trial was excluded from correlations to avoid detecting differences that may be due to predicted changes in CR feedback between conditions). Cell pairs showing positive correlations during standard training were compared with the same cell pairs after extinction, and the averages for each were calculated to compare across ensembles. The total number of positively correlated cell pairs was also calculated between conditions for each ensemble.

## Results

A persistent input in response to the CS is required to drive the cerebellar expression of trace CRs (Kalmbach et al., 2009; Chen et al., 2014). The current study investigates whether the modulation of persistent mPFC responses, as a necessary input to drive the cerebellar expression of trace CRs, mediates flexible learning to changing task demands. To this end, we recorded mPFC cells during the extinction and reacquisition of trace CRs, and during training to a different CS (Fig. 1). We hypothesized that the mPFC could mediate the extinction of CRs by decreasing persistent responses to the CS, which would preclude the cerebellar expression of CRs (Fig. 1*B*, top middle). In contrast, we hypothesized that persistent responses would generalize to the new CS during training to a different tone to facilitate rapid relearning to the new stimulus (i.e., animals would show savings; Fig. 1*B*, top right). However, previous work has shown that the mPFC receives feedback regarding the behavioral CR (Siegel and Mauk, 2013), and so any manipulation that changes behavior will cause some change in spiking in cells that receive such feedback in the current study. Therefore, we implemented two critical control analyses in testing the above hypotheses. First, we used a spike–eyeblink correlation analysis to identify which mPFC cells showed strong CR feedback responses^b^ and excluded those cells in follow-up analyses (see Materials and Methods). Figure 3 shows validation of the analysis for persistent cells compared with cells showing CR feedback alone in the absence of persistent responses (previously referred to as trace interval cells; Siegel et al., 2012; Siegel and Mauk, 2013). The second critical control was specific to extinction. We used temporary inactivation of the cerebellum with muscimol (Fig. 1*A*, pharmacological blockade) to abolish CRs as a non-learning-associated change in behavior, to compare with the learning-associated abolition of CRs via extinction training.

All cells were recorded primarily from layers 5/6 of the anterior cingulate or medial agranular regions of the mPFC that were previously shown to play a role in trace eyeblink conditioning (Fig. 2*A*; Kalmbach et al., 2009; Chen et al., 2014; Siegel et al., 2015). Standard training trials each day prior to behavioral manipulations were used to identify mPFC cells that showed persistent responses to the CS, as well as nonpersistent mPFC cells showing CR feedback responses as a readout of behavioral feedback to the mPFC (Fig. 2*B*; see Materials and Methods). Only well isolated single units (Fig. 2*C*) were included for analyses. Persistent cell recordings did not show systematic changes in isolation between standard and behavioral manipulations that could explain the observed changes in spike response^a^ (Fig. 2*D*; see Materials and Methods).

### Behavior during repeated extinction and reacquisition sessions

**Figure 4.**
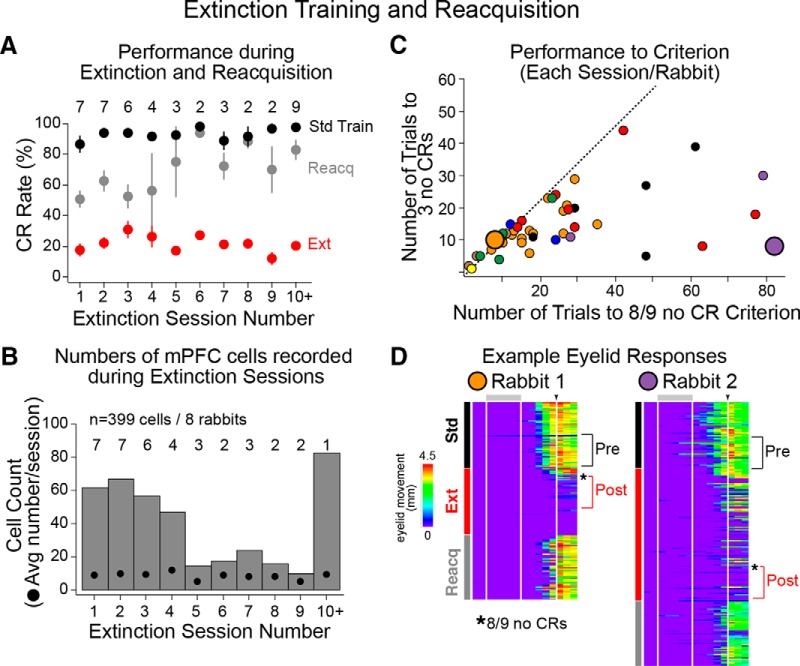
Behavioral performance during extinction sessions (standard training followed by extinction and then reacquisition of CRs) and behavioral criteria used to analyze spike data during extinction. ***A***, CR rates were used to assess changes in behavior between conditions and across weekly experiences. The average performance during standard (black markers) and extinction (red markers) training show decreased CR rates as a result of extinction training, and was similar across weekly experiences. Performance during reacquisition (gray markers) was lower and more variable between rabbits and across experiences than standard training (numbers indicate the number of extinction sessions each week). ***B***, Bar graph showing the total number of mPFC cells recorded during extinction sessions and the average number of cells/session recorded each week (black markers). The majority of cells were recorded during the first four extinction experiences. Numbers indicate the number of rabbits that experienced extinction for a given week. ***C***, Scatterplot showing the number of trials until there were three consecutive trials with no CR (*y*-axis, criterion for early extinction) and the number of trials to reach eight of nine trials with no CR (*x*-axis, criterion for late extinction; markers represent each session, color indicates specific rabbits). Sessions for which animals met both criteria at a similar time (near dashed identity line) suggest a rapid progress to full extinction, and typically occurred between 10 and ≥20 trials after the start of extinction training. Sessions in which criteria were met at different times (below identity line) suggest that the extinction process was slower. ***D***, Pseudocolored matrices of representative eyelid responses from two extinction sessions/rabbit (indicated in ***C*** by enlarged markers). One showed early rapid extinction (orange, left) and the other showed slow extinction (purple, right). Examples are from the same waterfall plots shown in Figure 1*A*. Brackets indicate trials used for the analysis of spike data to compare standard (last 27 trials) and full extinction based on when the eight of nine trials with no CR criterion was met (*first 27 trials after full extinction). Avg, average.

Rabbits displayed similar levels of performance during the standard training trials that preceded extinction training each week (Fig. 4*A*, black markers; 93.2 ± 1.2% CR rates for all pre-extinction standard training epochs). Animals also showed similar CR rates during extinction training across weeks (Fig. 4*A*, red markers; 21.8 ± 1.5% CR rates for all extinction epochs; but see Kehoe, 2006), while reacquisition training resulted in CR rates that were more variable and typically reflected poorer performance for the first three to four experiences (Fig. 4*A*, gray markers; 67.7 ± 3.5% CR rates for all reacquisition epochs). The activity of 399 well isolated unique single units was recorded in the mPFC of eight rabbits during extinction sessions (45 sessions; 8.87 cells/session). Animals also experienced reacquisition training for a majority of those sessions (40 of 45 sessions; 369 cells from eight rabbits). The average number of cells recorded during extinction sessions was similar across weeks (Fig. 4*B*, black markers; 8.9 ± 0.7 cells across all sessions). However, most mPFC cells were recorded during the first four extinction experiences (Fig. 4*B*, bar graph).

Although rabbits expressed similar overall CR rates during extinction each week (Fig. 4*A*), the number of trials to reach extinction criteria was highly variable across animals, and even across extinction sessions for a given rabbit (Fig. 4*C*, markers represent trials to criteria for each extinction session and are color coded for individual rabbits). Extinction behavior during some sessions was observed as relatively abrupt decreases in the expression of CRs, but was variable in the number of trials to onset (between 3 and 25 trials for most sessions, falling around the identity line in Fig. 4*C*). For other sessions, extinction was inconsistent and proceeded slowly, such that the number of trials until the first occurrence of three no-CR trials occurred relatively quickly (5–15 trials), but full extinction was not observed for 25–80 trials (Fig. 4*C*, markers falling below the identity line). Figure 4*D* shows behavioral examples of sessions in which rabbits showed faster (left) or slower (right) extinction (Fig. 4*C*, specific sessions indicated by large markers). Note that in either case rabbits showed relatively complete extinction after the eight of nine no-CR criterion was met (Fig. 4*D*, asterisks), and so the first 27 trials after this criterion was met were used to test whether persistent mPFC cells showed changes in activity in association with the extinction of CRs (i.e., during “postextinction” trials; Figs. 2*B*, top, 4*D*).

### Persistent mPFC cells do not show differences in CS-evoked responses after full extinction

For the majority of persistent cells, a persistent response pattern was observed across standard, extinction, and reacquisition training independent of how well a given rabbit extinguished or reacquired CRs (Fig. 2*B*, top left). In contrast, CR feedback cells typically showed decreases and increases in activity in accordance with the extinction and reacquisition of CRs, respectively (Fig. 2*B*, top right).

**Figure 5.**
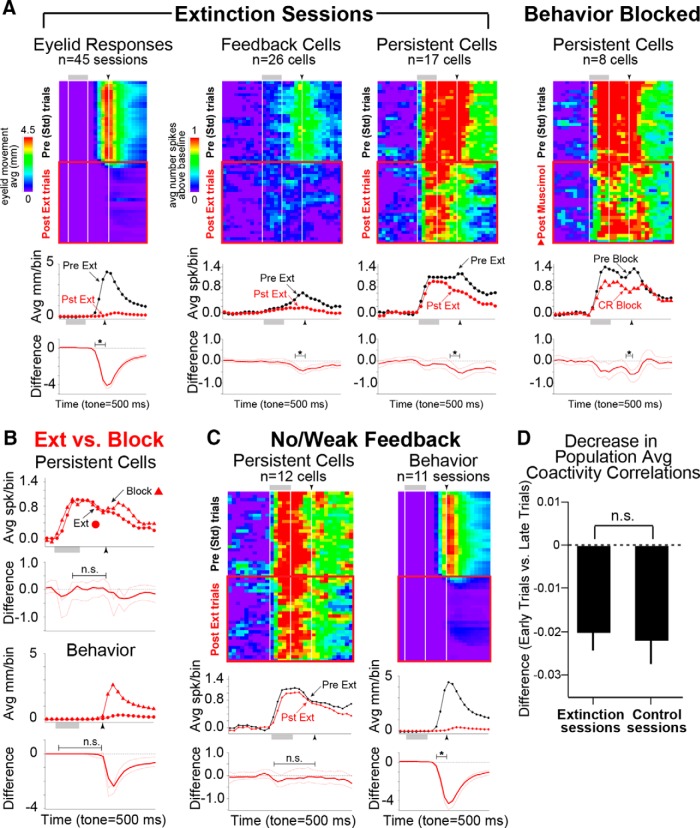
Persistent mPFC cells did not show decreased spike responses as a result of extinction training. Pseudocolored matrices and graphs are as described in Figure 3. ***A***, Comparisons between Std (black) and after full extinction (Pst Ext; red) for behavior (Eyelid Responses, left), CR feedback cells (middle), and persistent cells (right), and for sessions in which behavior was pharmacologically blocked by infusion of muscimol into the cerebellum (Behavior Blocked, far right). Pseudocolored matrices show eyelid position or spike activity during Std and Pst Ext trials, with each row showing eyelid or spike response from one trial, averaged across sessions or cells, respectively. Top graphs show averages from Std trials (black) and from Pst Ext or Post-muscimol infusion trials (red). Bottom graphs show bin-by-bin differences between Std and Post averages (red line) and confidence intervals from bootstrapping (dashed red lines) for each time bin. PFC cells only showed response changes in association with changes in behavioral feedback due to CR extinction, similar to that observed during pharmacological blockade of CRs, suggesting that learning-related changes were not observed. ***B***, Direct comparison of postextinction and pharmacological blockade of CRs indicated that observed changes in spike responses during Pst Ext trials were due to the absence of behavioral feedback alone and not to modification of responses to the tone as a result of extinction training. ***C***, Control comparisons for persistent cells identified as showing No/Weak behavioral feedback support the interpretation that persistent cells did not show changes in spike responses after extinction. The average behavior for sessions that contributed cells to this analysis is also given. ***D***, Correlated activity within simultaneously recorded cell ensembles show decreases in coactive cell pairs after extinction, which was similar to that observed during control sessions, suggesting that changes in ensemble activity occur over time and not in response to extinction training (unpaired *t* test, *p* > 0.05). Avg, average.

To test whether persistent cells showed response changes between standard and postextinction trials, raster plots were first binned across time and trials, and averaged across cells such that each row represents the average response of cells for each trial (Fig. 5*A*). A bootstrap procedure was used to test for differences in spike response for each trial time bin (Fig. 5*A*, bottom graphs show trial averages and bootstrap results). The same procedure was applied to eyelid responses, and demonstrated reliable decreases in eyelid responses (CRs) between standard and postextinction epochs^c^ (Fig. 5*A*, left; average CR rates: Std = 90.6 ± 1.6%, Ext: 12.0 ± 1.2%). The averaged binned raster plots of CR feedback cells and the corresponding bootstrap analysis demonstrated that full behavioral extinction resulted in reliable decreases in responses during the last three time bins (300 ms) of the trial, in accordance with the absence of CRs and behavioral feedback^d^ (Fig. 5*A*, center). A reliable decrease was also observed during the last 300 ms of the trial for persistent cells, but was not observed in earlier trial bins, suggesting that persistent cells did not show changes in response to the CS and only showed differences due to the absence of behavioral feedback^e^ (Fig. 5*A*, right). The same result was observed after pharmacological blockade of CRs^f^ (Fig. 5*A*, behavior blocked), in the absence of extinction learning. Persistent responses after extinction and during pharmacological blockade of CRs was directly compared to control for the absence of CRs and feedback between new learning (not to respond) and the simple absence of CRs (pharmacological blockade). No reliable differences in spike responses were observed between the two conditions^g^ (Fig. 5*B*), suggesting that any changes in spike response were due to the absence of CR feedback and not to changes in the response to the CS. As a final control, analysis was restricted only to persistent cells identified as receiving no/weak feedback^h^ (Fig. 5*C*). No reliable changes in persistent responses were observed for cells that did not receive CR feedback for any trial time bin, supporting the interpretation that persistent cells did not alter responses to the CS as a result of extinction training.

Although mPFC cells continued to show persistent responses throughout extinction, it is possible that changes in the precise patterns of response across cells within a population may result in net changes to cerebellar inputs and changes in behavioral output. For example, the histograms of the persistent cell shown in Fig. 2*B* (top left) suggest changes in the pattern of persistent response between standard and postextinction trials, even though the response still qualified as persistent. The correlated activity of pairs of cells within simultaneously recorded ensembles was compared to address this possibility (see Materials and Methods). Analysis was focused on the same ensembles from which persistent cells were recorded, for the same standard and postextinction trials used in the previous analyses. A significant difference in the number of coactive cell pairs between standard and postextinction trials was not observed for ensembles^i^ (10 ensembles with 8–14 cells/ensemble; Std, 8.6 ± 1.5 correlated cell pairs; Ext, 8.6 ± 1.0; paired *t* = 0.0, df = 9, *p* = 1.0). Significant decreases in the average correlations of cell pairs within ensembles were observed between standard and extinction trials^j^ (Std, *r* = 0.03 ± 0.004; Ext, *r* = 0.007 ± 0.001; paired *t* = 4.85, df = 9, *p* = 0.01). However, significant decreases were also observed for control extinction trials^j^ (Std Ctl, *r* = 0.03 ± 0.003; Ext Ctl, *r* = 0.01 ± 0.003; paired *t* = 4.03, df = 5, *p* = 0.01). The amount of decrease was not different between extinction and control sessions^k^ (Std – Ext, *r*_diff_ = 0.02 ± 0.004; Std Ctl − Ext Ctl, *r*_diff_ = 0.02 ± 0.006; unpaired *t* = 0.31, df_c_ = 10.38, *p* = 0.76; Fig. 5*D*), suggesting that changes in coactivity within ensembles occurs as a function of time and was not due to extinction training.

**Figure 6.**
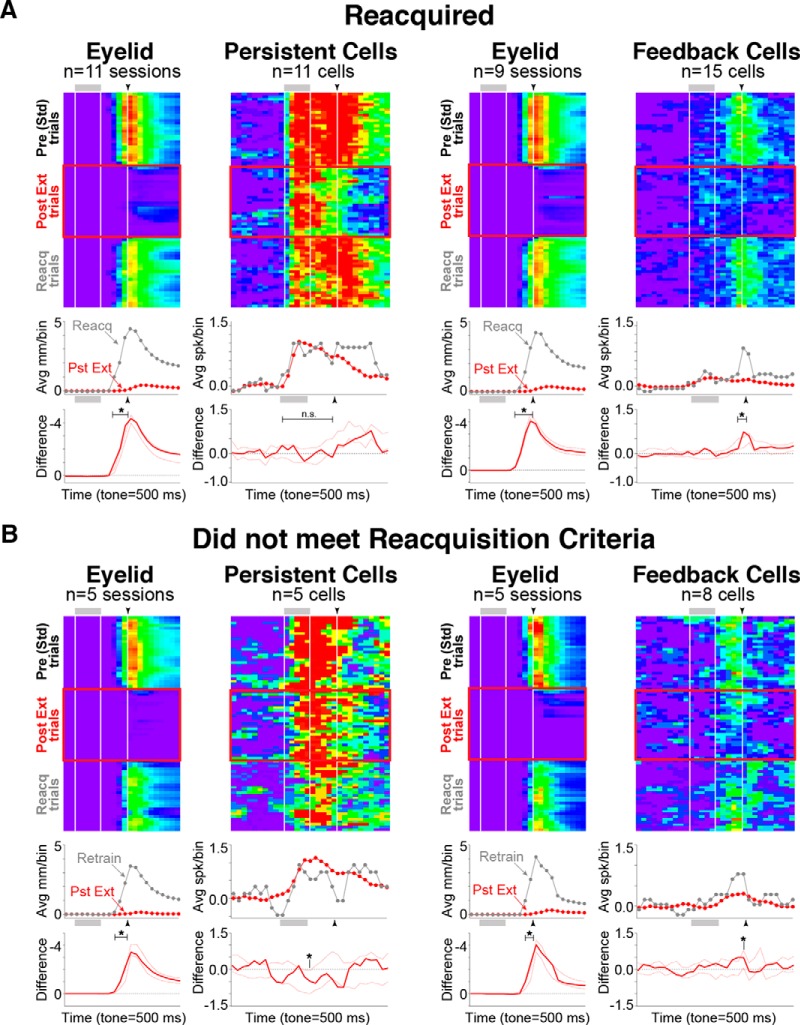
Persistent mPFC cells did not show changes in spike responses as a result of reacquisition training. Pseudocolor matrices and graphs are as described in Figure 5. Average spike activity from pre-extinction Std (black) is also shown for comparison. ***A***, Comparisons of behavior, and persistent and trace cell responses between postextinction (Pst Ext; red) and reacquisition (Reacq; gray). Reliable differences were not observed between Pst Ext and Reacq for persistent cells, while CR feedback cells showed reliable differences in spike responses in accordance with behavioral feedback. ***B***, Similar results were observed for persistent cells even when reacquisition criteria were not met. CR feedback cells showed more modest differences in responses when reacquisition criteria were not met, likely reflecting the behavioral variability observed for these sessions.

Changes in persistent responses were also not observed between postextinction and reacquisition trials, whether or not reinstatement criteria were met^l^ (Fig. 6*A*,*B*, left; reacquisition CR rates, 85.0 ± 3.5%). Feedback cells, however, did show a reinstatement of CR-associated responses with the reacquisition of learned responses^m^ (Fig. 6*A*,*B*, right; reacquisition CR rates, 80.8 ± 4.7%). Together, the data show that persistent mPFC cells did not show decreased responses to the CS as a result of extinction or reacquisition training, suggesting that the mPFC may not modulate the inhibition or reinstatement of this kind of learned behavior in such a straightfoward way.

### Behavior during different tone/interval sessions

**Figure 7.**
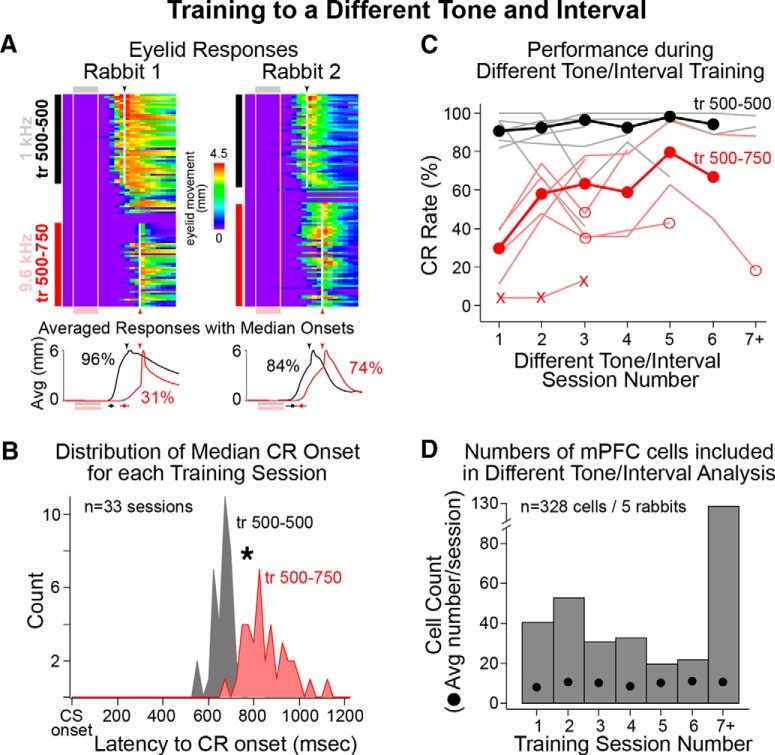
Behavioral performance during standard [1.3 kHz, training for 500–500 ms (tr 500–500), black] and different tone/interval training (9.6 kHz, tr 500–750, red), and analysis of CR onset latencies used to determine sessions in which rabbits showed good behavioral discrimination between the two tones. ***A***, Representative eyelid behavior from different tone/interval sessions from two rabbits. Sessions began with standard training, followed by 9–18 trials of extinction to facilitate the task switch to the different tone/interval. Line graphs show the average eyelid behavior for standard (black) and different tone training (red; CR rates also given). The latencies to CR onset were measured for each trial and tested between epochs (indicated by black and red markers below graphs; median latency ± quartile). Only sessions in which rabbits showed significant differences in the timing of CRs were included for spike analysis. Note that both rabbits showed significantly later CR onsets for trials with the different tone (red). ***B***, The distributions of median session latencies to CR onset are shown for standard (gray) and different tone/interval (red) epochs. A paired comparison indicated that rabbits showed significantly later CR onsets for different tone trials (paired *t* test, *p* < 0.001). ***C***, Average behavioral performance (CR rates) across weekly training sessions for standard (black markers, light black lines show individual rabbits) and different tone/interval training (red markers, light red lines show individual rabbits; “X” markers indicate sessions from one rabbit excluded for poor learning; open markers indicate sessions in which rabbits failed to discriminate between tones and so were excluded from spike analysis). Note that rabbits typically showed learning (increased performance) between the first two sessions of different tone training, but that CR rates were generally lower and more variable than those observed for standard training. ***D***, Bar graph showing the number of cells recorded during each weekly session, and the average number of cells recorded per session (black markers). Most cells included for analysis were recorded after rabbits showed new learning (post-session 1).

To test whether persistent activity in the mPFC could support flexible learning in response to a different stimulus, persistent cells were analyzed during standard training and training to a different tone. A longer trace interval was used in association with the different tone to ensure that animals were showing behavioral responses that were specific to new learning and the different stimulus. Two behavioral examples are shown in Figure 7*A* in which rabbits showed CRs with significantly different timing (latency to onset) between standard and different tone/interval training, demonstrating that they were discriminating between the different tones. Only sessions in which significant differences in the latencies to CR onset were observed, and therefore reflected behavior specific to the different tone, were included for analysis^n^ (Fig. 7*A*; 33 of 40 sessions from five rabbits; unpaired one-tailed *t* tests, *p* < 0.05; Fig. 2*B*, bottom for additional examples). The distributions of median latencies to CR onset for each standard and different tone/interval training session is shown in Figure 7*B*. A paired comparison of median latencies to onset during standard and different tone/interval training confirmed that rabbits discriminated between the two tones by shifting the timing of CRs^o^ (standard, 682 ± 9 ms; different tone, 866 ± 17 ms; paired *t* test, *t* = 14.11, df = 32, *p* < 0.001).

**Figure 8.**
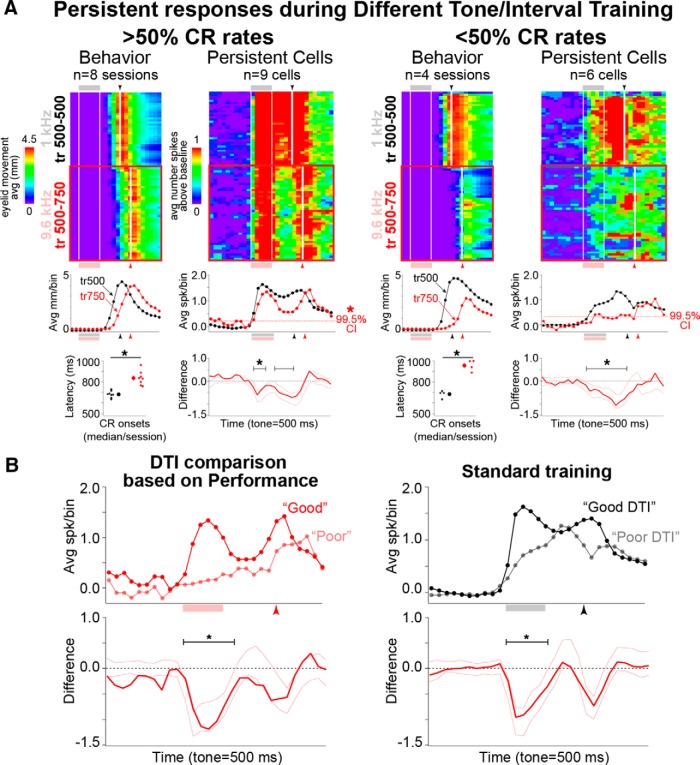
Higher performance (>50% CR rates) during different tone training was associated with generalized persistent responses, while poorer performance (<50% CR rates) was associated with decreases in persistent responses to near-baseline spike rates. Pseudocolored matrices and line graphs are as previously described. ***A***, Pseudocolored matrices of averaged eyelid behavior and persistent cell responses for different tone training in which rabbits showed >50% or <50% CR rates (good vs poor performance). Rabbits showed good behavioral discrimination between the two tones for good and poor performance sessions (bottom left plots, paired *t* tests, *p* < 0.001). Reliable decreases in persistent responses were observed during both good and poor performance sessions. However, mPFC cells maintained a persistent response pattern only when performance was >50% (left, red dashed line in top right graphs = upper confidence interval of bootstrapped baseline). Persistent responses approached baseline levels when performance was <50% (right). ***B***, Direct comparison of persistent cells during good and poor performance during different tone/interval training revealed reliable decreases in persistent responses when behavior was poor (left). Reliable differences were also observed during standard training for these cells, suggesting that weaker tone responses during standard conditions may predict decreased performance in response to the new task conditions.

Although rabbits showed reliable shifts in the timing of CRs between standard and DTI trials, the overall performance during different tone training was more variable across animals and between sessions than standard training (Std, 95.1 ± 1.0% CR rate; DTI, 64.9 ± 4.6% CR rate; Fig. 8*C*, open markers show the sessions excluded due to lack of behavioral discrimination, as described above). One rabbit showed little or no learning in response to the new tone over three sessions and was excluded from further analyses (Fig. 7*C*, sessions indicated by “X”). However, most rabbits showed learning during the very first training session in response to the new tone and interval, which was never observed when initially trained^p^ (first standard session, initial learning, 2.0 ± 2.0% CR rate, data not shown; first session different tone, 29.6 ± 5.3%; Fig. 7*C*; Wilcoxon signed rank test, df = 4, *p* = 0.03). The results suggest that experiencing the original learning facilitated the new learning (i.e., most rabbits showed “savings” in the new task; Napier et al., 1992; Weidemann and Kehoe, 2005). Rabbits also showed additional learning to the new tone as significant increases in performance between the first and second training experience^q^ (second session different tone, 58.0 ± 5.5% CR rate; one-tailed Wilcoxon signed rank test, df = 5, *p* = 0.03), after which there was no significant difference in performance between different tone sessions^r^ (63.3 ± 13.7% CR rate; session 2 vs 3: one-tailed Wilcoxon signed rank test, df = 2, *p* = 0.13; Fig. 7*C*).

For sessions with significant behavioral discrimination, rabbits met the performance criterion in 22 of 33 different tone/interval sessions. The average CR rate for those sessions was 80 ± 3.7%, indicating that animals typically performed well during sessions in which the criterion was met. For sessions in which the performance criterion was not met, CR rates varied between 11% and 48% (average CR rate, 34.8 ± 3.3%).

### Persistent mPFC cells showed generalized responses to the different tone associated with performance during different tone/interval training

A total of 328 cells (33 sessions from five animals) were included for spike analysis based on the ability of the animals to show behavioral discrimination between the standard and different tone CS (48 cells from seven sessions were excluded due to poor discrimination; Fig. 7*C*). Figure 7*D* shows the number of mPFC cells recorded during the different tone/interval sessions included for analysis. The average number of cells recorded during each session was similar across training days (Fig. 7*D*, markers). The recording sites of mPFC cells showing persistent responses are shown in Figure 2*A* [bottom (filled and open markers indicate recordings during good or poor performance, respectively)].

To test whether persistent mPFC cells show generalized responses to a different CS that could facilitate new learning and flexible behavior, the spike activity during standard and different tone/interval training was compared for sessions in which CR rates exceeded 50%. Persistent cells showed a decrease in spiking in response to the different tone/interval when performance was strong^s^ (Fig. 8*A*, left). Although decreased relative to standard training trials, bootstrapping analysis relative to pre-CS activity indicates that persistent cells maintained a persistent pattern of spiking that generalized to the different tone^t^ [Fig 8*A*, left (red dashed line indicates 99.5% confidence interval); see Materials Methods]. As further support, most of these cells still qualified as persistent when recategorized based only on different tone trials (seven of nine cells identified as persistent during standard training trials). Note that the shift in the timing of CRs was significantly different for the sessions in which these cells were recorded^u^ (paired *t* test: *t* = 6.76, df = 7, *p* < 0.001; Fig. 8*A*, leftmost graphs). For sessions contributing persistent cells with <50% CR rates during different tone/interval training, averaged eyelid responses showed less robust CRs that were, nevertheless, also shifted to longer latencies^v^ (paired *t* test: *t* = 19.45, df = 4, *p* < 0.001; Fig. 8*A*, right, behavior). Persistent cells recorded during these lower performance sessions also showed reliable decreases in spiking across trial bins^w^ (Fig. 8*A*, right, <50% CR rates). However, in contrast to persistent cells recorded during higher performance sessions, bootstrap analysis relative to pre-CS baseline activity revealed that these cells failed to maintain reliably persistent responses during DTI trials^x^ (Fig. 8*A*, right, red dashed line in top graph). When these cells were recategorized based on different tone trials, only two of six cells still qualified as being persistent. The results of analyses restricted to persistent cells receiving weak/no CR feedback was nearly identical, suggesting that the degraded responses of persistent cells, particularly during poor performance, was not simply due to a decrease or change in CR-associated feedback^y^ (data not shown). Direct comparison of persistent responses between the DTI trials of good and poor performance sessions was done to determine whether responses were indeed different. Bootstrapping analysis revealed a reliable difference between the responses of good and poor performers in response to the CS^z^ (Fig. 8*B*, left). Interestingly, direct comparison of persistent responses during pre DTI standard training indicated weaker persistent responses for sessions in which rabbits went on to show poor performance when switched to the different training conditions^a'^ (Fig. 8B, right), even though behavioral performance during the standard trials was indiscernible between the two groups (Fig. 8*A*, behavior, compare Std between right and left). The latter observation suggests that stronger persistent responses may be associated with an increased likelihood to generalize responses and support flexible task demands.

It should be noted that the observed performance-associated difference was not specific to persistent cells. Phasic cells also showed the same performance-associated effect^b'^ (data not shown), indicating that the entire mPFC network either generalized or failed to generalize in association with higher or poorer performance, respectively. The responses of CR feedback cells during DTI training directly reflected behavioral performance^c'^ (Fig. 2*B*, bottom right; group data not shown).

## Discussion

Previous work demonstrated that the persistent responses of mPFC cells lie upstream of the cerebellum (Siegel and Mauk, 2013), which is responsible for the generation of CRs (Kalmbach et al., 2009; Siegel et al., 2012; Chen et al., 2014). It is possible that the mPFC could mediate the behavioral expression of trace CRs via changes in the output of persistent cells. However, it was further demonstrated that many mPFC cells receive feedback regarding the cerebellar expression of CRs. Such reciprocity makes it difficult to disentangle whether changes in neural activity are potentially driving changes in behavior or are simply reflecting changes in behavioral feedback. Any manipulation that results in behavioral changes will be reflected as changes in feedback and will therefore result in changes in neural responses in the mPFC, and is a caveat to testing any relationship between neural activity and behavior.

**Figure 9.**
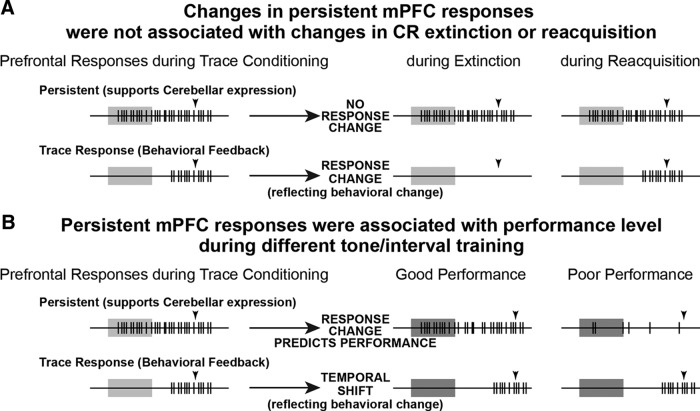
Schematic of the results for persistent and CR feedback cells during extinction and different tone training. ***A***, Persistent cells did not show a change in spike response after full extinction or during reacquisition, while feedback cells showed the predicted changes in association with the absence and reinstatement of CRs. The data suggest that a decrease in persistent mPFC responses is unlikely to mediate the extinction of CRs. ***B***, In contrast, during different tone/interval training persistent cells showed generalized responses when performance was good, while a failure to generalize was associated with poor performance. CR feedback cells showed a temporal shift in spike responses reflecting feedback from the shift in behavioral CRs.

The goal of the current study was to determine whether changes in the responses of persistent mPFC cells might drive flexible changes in behavior during trace eyeblink conditioning. Critically, the potential effect of changes in behavioral feedback were controlled for in the current study by (1) restricting analyses to cells identified as showing no/weak behavioral feedback in spike responses and (2) by comparing neural responses after the learning-related abolition of CRs (extinction) to the non-learning-related abolition of CRs (via pharmacological blockade). In the current study, persistent mPFC cells did not show altered responses as a result of extinction training (Figs. 5, 9*A*), suggesting that the mPFC may not mediate CR expression by decreased persistent responses or changes in the coactivity of cell ensembles when the same stimulus is used. Likewise, changes in persistent responses were not observed during reacquisition. In contrast, when rabbits were trained using a different tone and trace interval, the generalization of persistent mPFC responses to the different tone was associated with higher performance rates (Figs. 7, 9*B*). If persistent mPFC responses were weaker during standard training, a failure to generalize to the different tone was observed, with persistent responses decreasing to baseline levels in association with the poorer behavioral performance. Stronger persistent responses appear to result in an increased likelihood to generalize to a new stimulus and support changing task demands. The data suggest that decreases in persistent mPFC responses do not appear to mediate behavioral expression of the original learning, but may play a more direct role in the generalization of that learning to new or changing task demands.

### The role of the mPFC in extinction

Previous work suggested that there may be at least two mechanisms of extinction for trace conditioning in rabbits: one that is cerebellar and one that may be extracerebellar (Kalmbach and Mauk, 2012). One suggested extracellular mechanism was the inhibition or truncated responses of the mPFC cells that provide persistent inputs to the cerebellum in response to the CS. However, changes in persistent CS-evoked responses were not observed between expression and full extinction in the current study. Changes in the coactivity of cell ensembles as a result of extinction training also were not observed in the mPFC, perhaps suggesting that alternative extracerebellar mechanisms may be worth exploring. Interestingly, a previous study showed that lesions restricted to the more rostral mPFC disrupted the normal extinction of trace CRs (Weible et al., 2000), though acquisition and expression were spared. Those findings suggest that the rostral mPFC may play a role specific to the extinction of learned responses. Different regions of the mPFC play opposing roles in fear conditioning (Sierra-Mercado et al., 2011), and a similar scenario could mediate behavior in trace eyeblink conditioning as well, given that many mPFC regions converge onto a common cerebellar input pathway (Moya et al., 2014). A second alternative hypothesis is that cerebellar output may be inhibited during extinction in the red nucleus, which also has been previously suggested (Kalmbach and Mauk, 2012). The current study suggests that the simple and straightfoward idea that decreases in persistent responses may be a mechanism for extinction appears to be unlikely, and that future studies might focus on investigating alternative hypotheses.

Similar to previous reports, we did not observe systematic differences in the efficacy of repeated extinction experiences over several weeks (Kehoe, 2006). An interesting caveat to the current experiments is the idea that there is a transfer of the learned behavior from the hippocampus to the mPFC after acquisition (Takehara et al., 2003), and perhaps between regions of the mPFC several weeks after learning (Hattori et al., 2014). However, no differences were noted in the responses of persistent mPFC cells during extinction between the first 3 weeks postacquisition (half of the cells reported) and in those from cells recorded after that time period.

### The role of the mPFC in generalized learning

Persistent spiking in response to the CS is observed early in trace eyeblink conditioning (Siegel, 2014), and in the current study did not appear altered in response to extinction training. However, during changing task demands the failure of persistent mPFC cells to generalize to the different tone was associated with poor performance, and is the first report suggesting that mPFC cells could mediate flexible learning and behavioral expression in trace eyeblink conditioning. More specifically, the data indicate that the mPFC may play an important role in the generalization of previous learning to new learning, and that the neural basis of this ability lies in the generalized responses of mPFC cells to behaviorally relevant stimuli.

A critical implementation in the current experiment was to associate a different (longer) trace interval with the new/different CS, in order to ensure that the behavior was specific to the stimulus presented and that the animals were indeed discriminating between the two tones. Rabbits readily learned the new behavioral contingency and generally showed good discrimination when switching between task conditions. Interestingly, generalized mPFC responses were observed for most but not all sessions for a given animal. This may suggest that certain modulatory conditions must exist in order for generalized responses to occur, which sometimes fail. Additionally, response generalization appears to be a general property of the mPFC network because phasic cells also showed generalized responses to the new CS. A neuromodulatory influence in the mPFC could increase the likelihood of generalized responses to different inputs across the network, which may be acquired with experience during the different task or on occasion may fail, resulting in poor performance. The list of possible neuromodulators is extensive, but obvious candidates include acetylcholine and dopamine (Gulledge and Jaffe, 1998; Gao et al., 2003; Tseng and O'Donnell, 2004; Dembrow et al., 2010). Other possibilities include the modulation of metabotropic glutamate receptors (Sidiropoulou et al., 2009; Kalmbach et al., 2013) or modified network activity (McCormick et al., 2003).

### The role of behavioral feedback to the mPFC during trace eyeblink conditioning

The precise role of behavioral feedback in cortical circuits is currently unknown, but presents an important caveat when interpreting changes in spike responses during behavioral manipulations. This issue was addressed in the current study by identifying cells that showed strong or no/weak CR-associated feedback and analyzing those cells separately. These control analyses were critical for a strong interpretation of the results. In addition, the analysis also provided an initial assessment of the prevalence of feedback within this cortical network. Nearly half of trace cells (44%) and persistent cells (42%) showed strong CR-associated feedback. Interestingly, far fewer (only 15%) phasic cells showed strong feedback. It is presumed that phasic inputs from the mPFC are not necessary to support cerebellar learning in trace eyeblink conditioning (Kalmbach et al., 2009; 2010; Weiss and Disterhoft, 2011; Siegel et al., 2012). The data suggest that behavioral feedback in cortical circuits may be specific to cells that are playing an upstream role in the behavior, rather than being randomly dispersed across the local population. In this respect, persistent cells showing weak feedback may not be the cells supporting the behavior, and so control analyses restricted to those cells may actually be a caveat to the current interpretation. However, reliable differences were observed for cells receiving strong feedback only during the latter half of the trace interval, in association with the changes in behavioral responses, and did not represent a modification of neural responses to the CS. The function of CR-associated feedback to the mPFC during trace eyeblink conditioning remains elusive, as it has for other tasks and cortical regions. However, the trace conditioning paradigm provides a unique opportunity to study the role of behavioral feedback in cortical circuits because it can be conveniently blocked by cerebellar inactivation (Siegel and Mauk, 2013).

### Summary

The mPFC is known for its role in executive function and flexible behavior (Kolb, 1990; Devinsky et al., 1995; Fuster, 1997; Miller, 2000; Kesner and Churchwell, 2011). While this is a well accepted role for the mPFC across species, it is often difficult to define the nature of this role in animal models (Kolb, 1990; Kesner and Churchwell, 2011). We show here that learning not to respond and then reinstating the extinguished behavior is likely not mediated by a change in persistent firing in mPFC, while the generalization of previous learning to a new task does appear to be mediated by the mPFC (Fig. 9). Additional experiments are necessary to determine the source and mechanisms that support generalized responses in persistent mPFC cells. Nevertheless, the current findings represent an important step in understanding how the mPFC might support the flexible learning that is crucial to the survival of an organism.

## Synthesis

The decision was a result of the Reviewing Editor Kathryn Jeffery and the peer reviewers coming together and discussing their recommendations until a consensus was reached. A fact-based synthesis statement explaining their decision and outlining what is needed to prepare a revision is listed below. The following reviewers agreed to reveal their identity: Matthew Jones, David Bilkey

Your manuscript was assessed by two independent reviewers and myself, and we collectively concluded that the paper contains data of potential interest to behavioural physiologists but is in need of revision to increase clarity and rectify some of the conclusions, and it will need to be re-reviewed. The comments are summarized below.
